# Early maltreatment effects on adolescent attention control to non‐emotional and emotional distractors

**DOI:** 10.1111/ajpy.12139

**Published:** 2016-08-18

**Authors:** Paul Gray, Helen M. Baker, Gaia Scerif, Jennifer Y.F. Lau

**Affiliations:** ^1^ Department of Experimental PsychologyUniversity of OxfordOxfordUK; ^2^ NSW Department of Family and Community ServicesLiverpoolNSWAustralia; ^3^ Department of PsychologyKing's College LondonLondonUK

**Keywords:** adolescence, cognitive control, maltreatment, selective attention, threat bias

## Abstract

**Objective:**

Early maltreatment increases lifetime risk of psychopathology. Emerging models suggest that exposure to maltreatment leads to changes in cognitive processes associated with the processing of threat, including processes of selective attention. Existing data may be interpreted to suggest that maltreatment is associated with an automatic attentional engagement with threatening cues, or that maltreatment is associated with generally poorer attention control. Using a pair of attention tasks, this study sought to examine whether maltreatment was associated with threat‐related interference on attention processing and if this could be explained by poorer attentional control capacity.

**Method:**

Fifty‐one maltreated adolescents from an out‐of‐home care sample in New South Wales, Australia were recruited to complete two attention tasks. Data from 24 of these participants were compared with that of a sample of non‐maltreated peers matched on gender, age, cognitive ability, and household income to identify maltreatment‐associated group differences. Data from all participants were then used to explore the degree to which attention variables correlated with continuous measures of internalising symptoms, subtypes, and severity of maltreatment.

**Results:**

On the first task, maltreated adolescents showed significant interference from an irrelevant but non‐emotional distractor on reaction times when completing a central letter identification task under low perceptual task conditions. On the second task, maltreated adolescents also showed similar interference on a probe‐identification task that involved ignoring threatening (angry face) distractors, again under low perceptual load.

**Conclusions:**

These data may suggest difficulties exercising attention control following early maltreatment. These may contribute to the emergence of psychiatric disorders and other difficulties for those exposed to maltreatment.

**What is already known about this topic?**

- Maltreatment may disrupt key neurocognitive processes involved in the detection and processing of threat—which in turn may increase risks for psychopathology throughout the lifespan.
- Across various experimental tasks, children and adolescents who have experienced early maltreatment show disruptions in the attentional processing of threat.
- Children and adolescents who have experienced early maltreatment may also show more general reduced attention control capacity even when tasks are non‐emotional.

**What this topic adds?**

- While adolescents who had experienced maltreatment in childhood maintained equivalent accuracy on completing a simple probe‐identification task, they showed greater interference in their response times from irrelevant threat distractors when perceptual complexity of the task was low.
- These findings extended to interference associated with irrelevant non‐emotional distractors—though these findings may be weaker.
- Together these findings may suggest that when active control mechanisms are required to deploy effort away from distractors, maltreated adolescents struggle.

Maltreatment is a significant global problem: Exposure to early abuse and neglect is associated with a range of adverse outcomes, including greater risk of psychopathology across the lifespan (Green et al., 2010; Janssen et al., 2004; Scott et al., 2011). Models examining the processes by which exposure to childhood abuse and neglect confers risk for the development of psychopathology emphasise the apparent ‘calibration’ of the developing child to their lived environment, whereby these experiences shape neurocognitive development (Loman & Gunnar, 2010). That is, early exposure to stressful or threatening environments prompts the developing stress response system and associated neural networks, particularly the limbic system and frontal cortex, to be more responsive to threat‐related stimuli. In turn, this affects cognitive processing of threat (e.g., in attention and appraisal) and associated behavioural responses (e.g., approach and avoidance). In typical families, caregivers play a critical role in buffering the stress response system and scaffolding developing cognitive and behavioural skills and strategies to support a child's engagement with their environment. However, in families where there are potential sources of threat (for example physical abuse or domestic violence) or there is a failure to adequately buffer the stress response system through inattentiveness (neglect), children may be more likely to develop patterns of heightened threat responsivity. One domain in which this heightened responsivity to threat may emerge is in processes of attention, that is, the way individuals prioritise and attend to potential threats in their environment.

As well as documenting more general attention difficulties, such as in sustained attention, attention control, and visual‐spatial attention in children with a history of maltreatment (Beers & De Bellis, 2002; Bucker et al., 2012; De Bellis, Hooper, Spratt, & Woolley, 2009), studies have found that threat stimuli are preferentially attended to by those with a history of abuse and neglect. With two exceptions (Kelly et al., 2015; Pine et al., 2005), the majority of studies have reported facilitated attention towards threat‐related stimuli (Gibb, Schofield, & Coles, 2009; Pollak, Vardi, Putzer Bechner, & Curtin, 2005; Shackman, Shackman, & Pollak, 2007). For example, in one study (Pollak & Tolley‐Schell, 2003), a visual dot probe task was used to compare reaction times (RTs) to non‐emotional probes following the presentation of threatening versus neutral faces. Data showed that physically abused children were much quicker at detecting the probes that followed the threatening faces over the neutral faces suggesting that their attention was more engaged by the threatening face. Other data also support the interfering effects of threat on attention processing among maltreated children and adolescents (Cicchetti & Curtis, 2005; Curtis & Cicchetti, 2011; Pollak, Cicchetti, Klorman, & Brumaghim, 1997; Pollak, Klorman, Brumaghim, & Cicchetti, 2001; Pollak et al., 2005). In an event‐related potential (ERP) study, Shackman et al. (2007) found that maltreated children generated greater P3b amplitudes (an index of attention resource allocation) in response to angry, but not happy or fearful, targets than did controls.

One interpretation of these data is of an automatic attentional engagement with threatening cues among maltreated children and adolescents. Consistent with this explanation, these children and young people would selectively prioritise threat‐related stimuli for further processing. However, another interpretation is that maltreated children and adolescents have more generalised poorer attention control, i.e., a poorer ability to deploy effort away from distractors which is amplified in the context of disengagement from threat. The latter explanation would suggest a more general difficulty in the flexible use of attention away from distractors in general, but threat‐related distractors in particular. In the current study, we extended previous studies of attention biases for threat in maltreated youth by investigating whether these individuals also had difficulties deploying attention away from non‐emotional distractors (Task 1) and threatening distractors (Task 2). One approach to probing more general attention control mechanisms is to vary the demands of the primary task, for example, by presenting the probe under low perceptual load conditions (where perceptual identification is not difficult) and high perceptual load conditions (where perceptual identification is more demanding on attention) (Bishop, Jenkins, & Lawrence, 2007). According to perceptual load theory (Lavie, Hirst, De Fockert, & Viding, 2004), a task presented under high perceptual load conditions would challenge attentional resources to their limits and curtail the degree to which goal‐irrelevant distractors (threatening or not‐threatening) filter through into processing. However presenting the same task under low perceptual load conditions would require other active control mechanisms in order to maintain goal‐directed behaviour against the interfering effects of distractors. If these active attention control mechanisms were compromised in adolescents with a history of maltreatment, then distractors would be expected to interfere with the primary task under low but not high perceptual load conditions—an effect that would be expected to be amplified for threatening distractors.

To test these hypotheses that maltreated youth struggled to deploy attention control against non‐emotional and threatening distractors, we modified two existing experimental paradigms (Bishop et al., 2007; Pine et al., 2005). The first investigated the role of a non‐emotional distractor (a letter) under high and low load conditions. The second investigated whether the presence of a threatening distractor (an angry face and a fearful face) disrupted subsequent processing of a probe presented under high or low load conditions. Of note, we included angry faces consistent with prior work in this area, but were interested to see if the results extended to fearful faces too, which have been used extensively to assess attention biases in anxious children and young people (Dudeney, Sharpe, & Hunt, 2015). While our primary interest was in assessing group differences on the degree to which emotional stimuli disrupt attention processing, our long‐term goal is to identify factors that could increase risk for psychopathology. As attention biases have been linked to anxiety (Dudeney et al., 2015) and to some extent depressive symptoms (e.g., Platt, Murphy, & Lau, 2015), here we also assessed the association between any attention factor that differentiated maltreated and non‐maltreated adolescents with anxiety and depressive symptoms. We did this for all participants as well as for maltreated adolescents alone. Finally, as ‘maltreatment’ is not a homogenous factor but instead individuals vary on several dimensions including the type of maltreatment, chronicity and severity, such variables could have different attention phenotypes. For example, it has been suggested that difficulties in the executive functions that support attention control are more related to experiences of deprivation, while exposure to physical abuse and/or domestic violence may be more related to sensitivity to threat‐related cues such as angry facial expressions (McLaughlin, 2016). In addition Pine et al. (2005) found a significant negative relationship between maltreatment severity and attention bias for threat. In our final set of analysis, we therefore also investigated correlations between maltreatment‐related factors, specifically severity and subtypes on attention variables which differed between groups.

## METHODS

### Participants

Fifty‐one maltreated and 24 comparison adolescents were recruited from the greater Sydney area of New South Wales, Australia. Maltreated participants were recruited from the out‐of‐home care population in New South Wales where it had been deemed unsafe for them to remain at home. Comparison participants (the non‐maltreated group) were recruited through local parent networks in the same geographical region. Parents of comparison adolescents completed a brief screening interview to ensure their children had not been exposed to adverse childhood experiences. Because the maltreated and comparison participants were not matched for cognitive ability, we selected 24 maltreated participants from the larger maltreated sample who were matched to the comparison participants on gender, age, cognitive ability, and household income to conduct between‐group comparisons on our measures of attention. However, as we were also interested in investigating whether attention measures were associated with anxiety and depressive symptoms, particularly in the maltreated group, we retained all 51 participants for performing individual differences analysis. Ethical approval was gained through the University of Oxford and the NSW Aboriginal Health and Medical Research Council, overseeing additional protocols relating to the conduct of research where Aboriginal Australians are likely to be over‐represented. Aboriginal children are significantly more like to be in out‐of‐home care in Australia (Australian Institute of Health and Welfare, 2016), reflecting past policies of forced removals of Aboriginal children, intergenerational trauma and ongoing social disadvantage and marginalisation (Australian Human Rights Commission, 2015; Human Rights and Equal Opportunity Commission, 1997). This was reflected in our sample (Table 1). Efforts were made to increase the representation of Aboriginal children in the comparison sample; however, participants were not matched on the basis of cultural background. Permission for maltreated participants was granted through the NSW Department of Community Services as their legal guardian, as well their foster carers. Consent for non‐maltreated adolescents was granted by their parent/guardian. Young people were also asked to assent to their participation.

**Table 1:** Demographic details of the matched maltreated and non‐maltreated participants used in these analysis

	Matched maltreated	Matched non‐maltreated	Unmatched maltreated
Number (male)	24 (11)	24 (11)	27 (16)
Physical abuse	11 (46%)	—	14 (52%)
Sexual abuse	2 (8%)	—	2 (7%)
Neglect—failure to provide	20 (83%)	—	24 (89%)
Neglect—lack of supervision	17 (71%)	—	23 (85%)
Emotional maltreatment	18 (75%)	—	25 (93%)
Moral/Legal/Educational maltreatment	5 (21%)	—	4 (15%)
Age	13.51 (1.75)	13.90 (1.50)	13.56 (1.75)
Abbreviated battery SB‐V	98.79 (7.60)	100.62 (8.44)	88.44 (10.51)
Anxiety symptoms (STAIC)	13.76 (7.28)	12.5 (6.57)	13.56 (5.88)
Depression symptoms (CDI)	9.31 (6.69)	7.76 (7.90)	8.82 (4.93)
Caucasian	54%	75%	70%
Aboriginal	38%	25%	19%
Other/Not specified	8%	—	11%

*Note*. Unmatched maltreated participants who completed the study but who were not matched on age and IQ are included here for comparison purposes.

The maltreated group experienced at least one subtype of abuse and neglect but consistent with other studies, the majority of maltreated adolescents experienced three or more subtypes (62%, Table 1). The presence/absence and severity of the following subtypes: Physical abuse (PA), sexual abuse (SA), neglect—failure to provide (FTP), neglect—lack of supervision (LOS), emotional maltreatment (EM), and moral, legal or educational maltreatment (MLE) were coded from file records using the Maltreatment Classification Scheme (MCS) (Barnett, Manly, & Cicchetti, 1993). Specifically, each notification was coded for each maltreatment subtype and the severity of the abuse/neglect using 0 to indicate absence and 1–5 to indicate severity (Garrido, Taussig, Culhane, & Raviv, 2011; Litrownik et al., 2005). Overall presence or absence of a given subtype of a particular participant reflected a coding of at least 1 across all notifications received. One quarter of all maltreated participants records were coded independently by two researchers (PG and JL). Inter‐rater reliability for maltreatment subtype presence or absence was calculated using kappas (all = 1.0, except FTP and EM which were unable to be calculated due to the absence of variance between coders). Inter‐rater reliability for maximum severity was also high (ICCs > =0.615).

Maltreated adolescents in this sample had been in care (i.e., removed from their maltreating home environment and placed into statutory foster care), and in their current placement, for a significant period of time (mean 9.4 and 7.3 years, respectively), despite generally experiencing a number of placement changes (mean 3.4).

### Measures

#### *Demographics*

Gender, age, and household income were all completed by the carer or parent.

#### *Cognitive ability*

To assess cognitive ability, all participants completed the two subtests (verbal knowledge and non‐verbal fluid reasoning) of the Abbreviated Battery of the Stanford–Binet Intelligence Scales—Fifth Edition (SB‐V). This was administered and scored by a registered psychologist (PG). The SB‐V reports appropriate reliability and validity.

#### *Anxiety symptoms*

Children completed the trait version of the Spielberger State‐Trait Anxiety Inventory for children (STAIC) (Spielberger, 1973). The STAIC is a widely used 20‐item self‐report measure, asking children to indicate whether a particular statement describes them *hardly ever*, *sometimes*, or *often*, which are then summed to create an overall anxiety score.

#### *Depression symptoms*

The Children's Depressive Inventory (CDI; Kovacs, 1985) was used to measure depressive symptoms in children. The CDI is a 27‐item self‐report measure, asking children to indicate which statement best describes them (i.e., *I am sad once in a while*, *I am often sad*, and *I am sad all the time*). An overall depressive symptoms score is then calculated.

#### *Non‐emotional attention control task*

**Figure 1:**
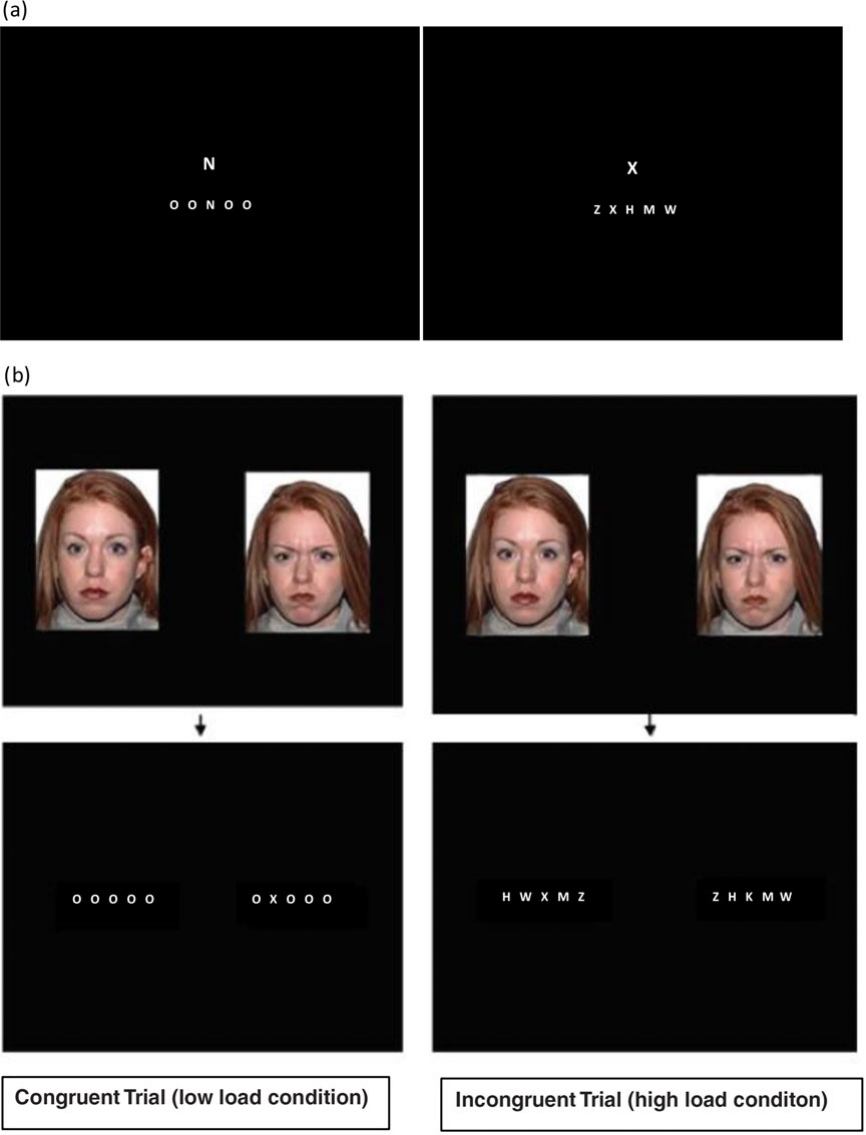
(a) A schematic of the non‐emotional attention control task. Participants were instructed to identify whether the letter string contained an X or N, with the letter appearing above the letter string as a congruent or incongruent distractor. A congruent distractor would be one where the letter matched the target letter that appeared in the letter string (e.g., an X above a letter string containing an X), whereas an incongruent trial occurred when the distractor letter was different to the target letter appearing in the letter string (an N above a letter string containing an X). The first screenshot illustrates a congruent low perceptual load trial while the second shows a congruent high perceptual load trial. (b) A schematic of the modified visual probe task. A congruent trial was one in which the target letter appeared hidden in the letter string in the place of the threatening face whereas an incongruent trial was one in which the target letter appeared in the letter string following the neutral face. These screen shots also illustrate the perceptual load conditions.

This task, which was presented on a laptop computer using E‐Prime, assessed attention interference to a non‐emotional distractor. First, a fixation cross was displayed in the centre of the display for 500 ms. This was replaced by a string of five letters, above which a task‐irrelevant distracting letter (either X or N) displayed in a larger font was presented (Fig. 1a). Participants were asked to indicate whether the target string included an ‘X’ or an ‘N’ by pressing the corresponding key on the keyboard, as quickly and accurately as possible. There were two conditions in this task. Congruent trials were those in which the distracting letter and the target letter in the string below were the same. Incongruent trials were those in which the distracting letter and the target letter in the string below were not the same. Trials also varied with the perceptual load of the letter string in which the target was embedded. Under high perceptual load conditions, the target was ‘hidden’ amongst a string of similar distractors (H, K, M, W, or Z). In the low perceptual load condition, the target was placed within a string of dissimilar, uniform distractors (O's). There were 40 trials for each level of load (80 trials total). The position of the target letter in the string was randomised for each trial. RT and accuracy was recorded on each trial.

#### *Modified visual probe task*

This task, which was also presented on a laptop computer using E‐Prime, assessed attentional interference to a threatening stimulus. A threatening stimulus was represented by an adult face displaying an angry or fearful expression consistent with prior studies. Faces were drawn from the NimStim^1^ set of expressions (10 models and 5 female). In a typical visual probe experiment, participants are asked to identify the location (left and right) of a visual probe presented on screen, immediately after the presentation of two simultaneously presented stimuli where one is a threatening stimulus and one which is a non‐threatening stimulus. Because we were interested in interference on a post‐threat task, in our variant of the task, participants were asked to identify on which side a target letter appeared, while hidden within a letter string. Additionally, we manipulated the degree to which the probe was more or less easy to identify by presenting high and low perceptual load conditions. The trial began with a fixation cross presented centrally for 500 ms. This was then replaced by two images each depicting the face of a single model presented equidistant from the centre point, to the left and right for 500 ms. In our task, one of the two images was always threatening (angry or fearful face) and the other was always non‐threatening (neutral face)—and the appearance of each image on the left or right was counterbalanced across trials. After 500 ms, the images were replaced by two strings of five letters, one on the left and one on the right. Participants were asked to identify which the location of the letter string that contained the target letter X by pressing the appropriate key; ‘Z’ for the left string, and ‘M’ for the right string, as quickly and accurately as possible. Trials varied with regard to whether the target letter appeared in the same location as the threatening stimulus (angry and fearful faces) or the non‐threatening stimulus (neutral face). Congruent trials were those where the letter string containing the target appeared on the same side as the threatening stimulus. Incongruent trials were those where the target appeared on the opposite side as the threatening stimulus. While previous studies of the attention disruptions amongst maltreated children and young people have used angry faces (Pollak & Tolley‐Schell, 2003), in the anxiety literature, fearful faces are often used (e.g., Ladouceur et al., 2009). Both angry and fearful faces were therefore included to test whether attention biases in maltreatment occur to angry faces as reported in previous studies—and whether these extended to fearful faces.

Trials also varied with the perceptual load of the letter string in which the target was embedded. Perceptual load was manipulated in the same way as the non‐emotional attention control task. Under high perceptual load conditions, the target was ‘hidden’ amongst a string of similar distractors (H, K, M, W, or Z). In the low perceptual load condition, the target was placed within a string of dissimilar, uniform distractors (O's). The target letter (X) was randomly placed in one of the three central positions in each trial. The Load condition was presented block‐wise, with order (High Load first or Low Load first) counterbalanced across participants.

A summary of the task and different trial types is presented in Fig. 1b. In summary, individual trials differed on the basis of congruency, the specific threatening face used (angry/fearful), and the level of perceptual load of the letter string in which the target appeared (high and low). There were 20 trials of each type (angry congruent, angry incongruent, fearful congruent, fearful incongruent, angry only, and neutral only) amounting to 120 trials for each level of perceptual load. Additional trials showed either two angry or two neutral faces to test whether there were group differences in RTs to threatening and non‐threatening faces. No group differences emerged. Participants also completed five practice trials in order to ensure that they understood the task. RT and accuracy was recorded on each trial.

#### *Analysis*

Measures of interest in each task included accuracy and RT data. Accuracy scores for each trial type were calculated, denoting the proportion of trials in which the respondent accurately indicated the position or nature of the probe. Mean RT scores were also calculated for each trial type in each task, based on valid RT data. Valid RT data in each task were those trials in which the probe position was accurately identified, and the response was made slower than 200 ms and within two standard deviations of each participant's mean response time for that level of load, consistent with other studies of this type (Pine et al., 2005). Where mean RTs were skewed, analyses were completed on log‐transformed data.

All between‐group differences analysis was performed on the 24 matched maltreated and 24 non‐maltreated adolescents. Data from the 27 maltreated adolescents who were not included in the between‐group analysis because of the need to match groups on age, gender, IQ, and family income, are nonetheless included in Tables for comparison purposes (i.e., to show that the pattern of results characterising the matched maltreated adolescents was characteristic of the maltreated group as a whole). For the non‐emotional attention control task, separate 2 × 2 × 2 mixed ANOVA with condition (congruent/incongruent) and load (low/high) as within‐subject factors, and group (maltreated/non‐maltreated) as a between‐subjects factor were performed on accuracy and RT data. For the modified visual probe task, again separate 2 × 2 × 2 mixed measures ANOVA were conducted with condition (congruent/incongruent) and load (low/high) as within subject factors, and group (maltreated/non‐maltreated) as a between subjects factor on accuracy and RT data. However , angry and fearful trials were analysed in separate analysis because power to detect higher‐order four ‐way interactions was limited.

In order to assess whether maltreatment‐linked differences in attention performance across these tasks may reflect latent vulnerability factors for psychopathology, we assessed correlations between any attention measure that differentiated the maltreated and non‐maltreated adolescents and anxiety and depressive symptom measures. We calculated these correlations for all the participants (including the non‐matched maltreated adolescents , so that total sample size for these analysis was 75 due to there being 51 maltreated adolescents and 24 comparison adolescents) as well as for the maltreated adolescents alone (*n* = 51) and non‐maltreated adolescents alone (*n* = 28) using summary attention bias and interference scores. Summary attention bias scores from the visual‐probe task were calculated by subtracting congruent RTs from incongruent RTs in each load condition separately, while summary interference scores from the non‐emotional attention control task were also calculated as by subtracting congruent scores from incongruent scores in each load condition separately. In order to assess whether maltreatment subtypes differentially effect attention bias and interference scores, we conducted two one‐way ANOVAs with maltreatment subtype as the between‐subjects factor (comparing those with neglect only vs those with a combination of neglect and physical abuse, as this was the only division of participants into distinct subtypes that included enough participants in each group). In addition, to establish whether the severity of maltreatment subtypes predicted attention bias and interference scores we calculated correlations between neglect severity scores and physical abuse severity scores with attention bias scores and interference scores in the maltreated sample (*n* = 51). If necessary, outliers were removed and where symptom or severity scores were skewed, analyses were completed on log‐transformed data or equivalent non‐parametric tests were used.

## RESULTS

### Non‐emotional attention control task

Accuracy and RT data for this task are presented in Table 2. There were no observed congruency, load, or group effects (all *p* = n.s.) on accuracy suggesting equivalent performance across conditions and participants.

**Table 2:** Mean (standard deviation) reaction times (in ms) and accuracy rates (in %) for congruent and incongruent, high, and low trials on the non‐emotional attention control task

	Matched maltreated		Matched non‐maltreated		Unmatched maltreated	
	Congruent	Incongruent	Congruent	Incongruent	Congruent	Incongruent
Reaction time						
Low load trials	681.92 (147.94)	717.16 (168.19)	601.56 (130.82)	607.90 (143.01)	733.78 (161.09)	779.60 (226.00)
High load trials	1345.98 (455.39)	1413.19 (488.04)	1195.81 (315.57)	1168.02 (331.08)	1547.31 (626.10)	1585.81 (580.43)
Accuracy						
Low load trials	0.90 (0.10)		0.92 (0.05)		0.90 (0.08)	
High load trials	0.90 (0.10)		0.92 (0.07)		0.91 (0.07)	
Overall	0.90 (0.09)		0.92 (0.05)		0.91 (0.07)	

RT data revealed a main effect of Load (*F*(1, 46) = 1065.467, *p* < 0.001, partial eta = 0.959) with participants responding more quickly in the low load condition. A main effect of group was also observed (*F*(1, 46) = 4.073, *p* < 0.05, partial eta = 0.081), with maltreated participants responding in general more slowly than their non‐maltreated peers. All other main and interaction effects were not significant (*p* = n.s.).

Because the main effects of group on RTs might mask more subtle differences between conditions within groups (including our a priori interest in the effects of a distractor under low load conditions in the maltreated group), we conducted some post‐hoc analysis on scaled ‘interference’ scores. More specifically, we calculated the scaled interference scores as the difference between RTs for incongruent and congruent trials (for each load) and re‐expressed this as a proportion of the total congruent and incongruent RT for each individual. Each of these scaled interference scores were compared to 0 using a one‐sample *t*‐test. Any significant deviation from 0 suggested significant interference, with positive scores reflecting greater interference. These data showed that maltreated participants had a significant interference score in the low perceptual load condition (*t*(23) = 2.208, *p* = 0.037).

A non‐scaled summary interference score (calculated by subtracting congruent scores from incongruent scores) under low perceptual load conditions did neither correlate significantly with anxiety (*r* = 0.113, *p* = 0.336, two‐tailed) and depressive symptoms (*r* = 0.129, *p* = 0.274, two‐tailed) in all participants—nor in the maltreated group alone for both anxiety (*r* = 0.121, *p* = 0.404, two‐tailed) and depression (*r* = 0.029, *p* = 0.839, two‐tailed) symptoms. We also assessed differences between maltreatment subtypes and low load interference scores. Specifically comparing adolescents with only neglect and those with a combination of physical abuse and neglect showed no significant group differences. Within the maltreated group, a non‐significant correlation was found between interference scores and the severity of neglect (*r*_s_ = −0.111, *p* = 0.425, two‐tailed) and the severity of physical abuse experienced (*r*_s_ = −0.117, *p* = 0.420, two‐tailed), indicating that the severity of each maltreatment subtype is not a predictor of interference scores.

### Modified visual probe task

Accuracy and RT data for this task are presented in Table 3. Although analyses on accuracy were repeated for angry and fearful trials separately both showed similar results: only a significant main effect of load emerged (*F*(1, 46) = 31.704, *p* < 0.001, partial eta = 0.408 and *F*(1, 46) = 19.175, *p* < 0.001, partial eta = 0.294) for the analysis with angry vs fearful faces, respectively) suggesting more errors on the high perceptual load condition. However, no significant main or interaction effects involving group and congruency emerged (all *p*‐values > 0.15) suggesting equivalent performance in terms of accuracy across individuals.

**Table 3:** Mean (standard deviation) reaction times (in ms) and accuracy rates (in %) for congruent and incongruent, high and low, and angry and fearful trials on the dot probe task

	Matched maltreated		Matched non‐maltreated		Unmatched maltreated	
	Congruent	Incongruent	Congruent	Incongruent	Congruent	Incongruent
Dot probe task, average reaction time						
Low load, angry trials	566.08 (146.48)	584.46 (163.96)	467.17 (69.80)	468.69 (64.39)	612.49 (172.57)	627.42 (200.04)
High load, angry trials	978.72 (304.51)	971.09 (325.15)	795.39 (205.21)	833.02 (239.06)	1115.61 (469.26)	1169.38 (433.45)
Dot probe task, accuracy						
Low load, angry trials	0.97 (0.04)	0.98 (0.03)	0.98 (0.04)	0.98 (0.03)	0.98 (0.04)	0.97 (0.041)
High load, angry trials	0.92 (0.08)	0.93 (0.05)	0.94 (0.6)	0.95 (0.06)	0.93 (0.07)	0.93 (0.08)
Dot probe task, average reaction time						
Low load, fearful trials	590.76 (218.74)	589.31 (187.09)	472.43 (76.70)	471.26 (61.97)	589.12 (115.58)	599.93 (135.03)
High load, fearful trials	970.96 (309.05)	964.26 (270.13)	781.55 (170.11)	788.12 (194.69)	1133.91 (505.32)	1147.87 (436.42)
Dot probe task, accuracy						
Low load, fearful trials	0.98 (0.04)	0.96 (0.04)	0.97 (0.04)	0.96 (0.05)	0.96 (0.05)	0.97 (0.05)
High load, fearful trials	0.93 (0.06)	0.93 (0.07)	0.94 (0.08)	0.94 (0.06)	0.92 (0.07)	0.93 (0.07)

In the analysis of the RTs for angry trials, main effects of load (*F*(1, 46) = 316.98, *p* < 0.001), congruency (*F*(1, 46) = 4.07, *p* = 0.049), and group (*F*(1, 46) = 7.794, *p* = 0.008) emerged. Responses were quicker in the low load condition than the high load condition. Congruent trials were also faster than incongruent trials suggesting an attention bias towards angry faces in all participants. Maltreated participants were slower than their non‐maltreated peers. Beyond main effects, there was only a significant load × congruency × group interaction (*F*(1, 46) = 5.22, *p* = 0.027). Analysing the load and congruency effects in each group separately did not yield significant interactions in either. Therefore, again given the main effects of group on RTs might mask any more subtle differences between conditions within groups (including our a priori interest in the effects of the angry face under low load conditions in the maltreated group), we conducted some post hoc analysis on scaled ‘bias’ scores comparing them against a no bias score (0). Scaled bias scores here were calculated as the difference between the congruent and incongruent trials, expressed as a proportion of the total congruent and incongruent trial RT for that individual. Analysing these data showed that maltreated participants had a significant bias score for threatening faces (i.e., their RTs to congruent trials were significantly faster than incongruent trials resulting in a negative difference score that was significantly different from 0) only when the perceptual load was low (*t*(23) = 2.54, *p* = 0.018) but not high (*t*(23) = −0.456, *p* = 0.653). Unexpectedly, non‐maltreated participants had a significant bias score for threatening faces when the perceptual load was high (*t*(23) = 2.375, *p* = 0.026) but not low (*t*(23) = 0.479, *p* = 0.637).

Similar analyses were conducted for RTs to fearful facial expressions. Again, there were main effects of load (*F*(1, 46) = 332.892, *p* < 0.001), and maltreatment group (*F*(1, 46) = 9.306, *p* = 0.004) but not of congruency (*F*(1, 46) = 0.021, p = 0.886).

We investigated the degree to which attention biases for angry faces under low perceptual load correlated with anxiety and depressive symptoms in the entire sample and in the maltreated group alone. Non‐scaled summary attention bias scores were calculated by subtracting congruent RTs from incongruent RTs. The correlation between anxiety symptoms and attention bias scores was non‐significant for both the entire sample (*r* = −0.212, *p* = 0.072, two‐tailed) and the maltreated group alone (*r* = −0.243, *p* = 0.092, two‐tailed). The correlation between depression symptoms and attention bias scores was not significant for the entire sample (*r* = −0.225, *p* = 0.056, two‐tailed). In contrast, the correlation between depression symptoms and attention bias scores for the maltreated group alone was significant (*r* = −0.358, *p* = 0.012, two‐tailed), such that greater depression symptoms predicted an attentional bias away from angry faces in this group (under low perceptual load). We examined differences between maltreatment subtypes and low load attention bias scores; specifically comparing adolescents with only neglect and those with a combination of physical abuse and neglect. However, no significant group differences were found. Within the maltreated group, a non‐significant correlation was found between attention bias scores and the severity of neglect (*r*_s_ = 0.111, *p* = 0.446, two‐tailed) and the severity of physical abuse experienced (*r*_s_ = 0.151, *p* = 0.299, two‐tailed), indicating that the severity of each maltreatment subtype is not a predictor of attention biases.

Because non‐maltreated adolescents showed an attention bias for the angry face under high perceptual load conditions, we also assessed correlations with symptom scores across the entire sample and for the non‐maltreated group alone. The correlation between anxiety symptoms and attention bias scores was not significant for both the entire sample (*r* = −0.058, *p* = 0.633, two‐tailed) or the non‐maltreated sample alone (*r* = −0.109, *p* = 0.582, two‐tailed). Likewise, the correlation between depression symptoms and attention bias scores was non‐significant for both the entire sample (*r* = −0.114, *p* = 0.345, two‐tailed) and the non‐maltreated sample alone (*r* = −0.173, *p* = 0.379, two‐tailed)

## DISCUSSION

This study extended previous studies of selective attention in maltreated youth by investigating more generalised attention control mechanisms when presented with non‐emotional and threatening distractors. Our results suggest that while maltreated adolescents showed equivalent levels of accuracy in performing the tasks, more subtle differences in processing capacity emerged in RT data. Specifically, data from both tasks indicated that when maintenance of a goal (identifying a target letter or its location) is modulated by an individual's attentional capacity rather than being exhausted by task demands (i.e., under conditions of low perceptual load), maltreated adolescents showed greater interference by both a task‐irrelevant non‐emotional and threatening distractor. In contrast, when attentional capacity was fully absorbed by a more perceptually complex task (i.e., high perceptual load conditions), leaving less room for individuals’ attentional capacity to influence task performance, maltreated adolescents were not distracted by the non‐emotional or threatening distractor. Unexpectedly, however, non‐maltreated adolescents did show significant interference of task performance by the threatening face in the visual probe task under high perceptual load conditions. Finally, within the maltreated sample, those with higher levels of depressive symptoms showed greater attention‐avoidance of angry faces in the low perceptual load condition of the visual‐probe attention‐orienting task. There were no differences between two subtypes of maltreatment (neglect only vs neglect and physical abuse) on attention variables—and the severity of the early abuse/neglect was also not associated with attention variables.

Our findings relating to maltreated adolescents suggested that they had significant difficulty exercising attention control to prevent the interference of this task‐irrelevant stimuli in general, whether emotional or not. According to a perceptual load theory of selective attention (Lavie, 1995; Lavie, 2005; Lavie et al., 2004), under high perceptual load conditions, further processing of task‐irrelevant stimuli (non‐emotional as well as emotional) is filtered out early, allowing cognitive resources to remain focused on the task at hand. Under the low load condition, cognitive capacity is not exhausted allowing task‐irrelevant cues to be processed, and therefore requiring the exercise of attention control to maintain task performance and prevent the interference of these cues. In the maltreated adolescents, to maintain accuracy on the central task, these difficulties of attention control were apparent through slower RTs on trials where there was an incongruent albeit non‐emotional distractor (Task 1) and on trials following an angry distractor face (Task 2). This poorer ability to exercise effortful processes of attention to maintain task performance are consistent with prior work measuring more general attention difficulties as measured by tasks such as the Stroop task and the Digit Vigilance task (Beers & De Bellis, 2002; Bucker et al., 2012; De Bellis et al., 2009). These difficulties may also provide a more basic explanation of previous reports of attention‐orienting or attention capture by threat in these individuals. It is interesting to note some degree of specificity in the bias. Notably, these findings emerged to angry faces (as reported previously by other studies) rather than to fearful faces which have usually been found to provoke attention biases in children and young people with anxiety (Dudeney et al., 2015).

Difficulties of attention control, which could potentially facilitate hyper‐vigilance to or even hyper‐avoidance away from threat cues may operate as a ‘latent vulnerability’ conferring risk of psychopathology for maltreated adolescents (Mccrory & Viding, 2015). Indeed, our data suggested that within the maltreated sample, high levels of depressive symptoms were associated with greater attention‐avoidance away from angry faces particularly under conditions whereby there were less demands on attention resources. Thus, the neurocognitive calibration of the attention processing system reflecting the early threatening (physical abuse and/or exposure to domestic violence) or unpredictable and unregulated (neglectful) developmental environment instantiates vulnerability over the longer term with there being a long‐term ‘cost’ to neurocognitive processes designed to promote short‐term safety and survival. Our data were consistent with the suggestions made by McCrory and Viding (2015) that altered threat processing may be one exemplar of latent vulnerability. Evidence from developmental anxiety literature identifies similar threat‐related attention biases as a precipitating and maintaining factor for the development of anxiety and depression with some suggestions that this can be hypervigilance for threat—but on some occasions, attention‐avoidance emerges too (see Shechner et al., 2012 for a review of paediatric anxiety studies and Platt, Waters, Schulte‐Koerne, Engelmann, & Salemink, 2016 for a review of child and adolescent depression studies). Longitudinal studies will be needed to clarify whether neurocognitive calibrations associated with early abuse/neglect do in fact contribute to the emotional difficulties experienced by maltreated children and young people across the life course. Understanding processes of latent vulnerability will allow the development of targeted interventions designed at preventing maladaptive outcomes before they emerge, rather than responding to symptom onset as is currently common (Mccrory & Viding, 2015)—and is therefore critical to improving the long‐term outcomes of maltreated children and young people and reducing social costs.

An unexpected finding emerged relating to attentional interference by a threatening distractor among non‐maltreated adolescents but under high perceptual load conditions. This finding warrants particular explanation as it has implications for the validity of our task and whether the two conditions in fact posed low and high perceptual demands on attention. This finding that non‐maltreated adolescents showed interference from an angry face in the high perceptual load condition is surprising, given that we expected this condition to be sufficiently absorbing of attention resources that irrelevant threat stimuli would not be able to filter through. One factor that could increase the interfering effects of threat on the central task of target identification is if this condition was cognitively taxing. In a series of experiments, Lavie et al. (2004) demonstrated that unlike high perceptual load conditions, tasks that were cognitively demanding (e.g., used working memory) actually increased distractor interference. It is possible that in healthy non‐maltreated adolescents, the ability to suppress distraction on this task broke down when they were also challenged by another demanding activity. It is not clear why this would not apply to the maltreated group but speculatively, it may be that as these participants had lower overall attention control and capacity, this task only posed perceptual demands rather than engaged working memory resources as well.

This study must be considered in light of relevant limitations. While a notable strength of our study was that participants in the maltreated and non‐maltreated group were matched on age, IQ, and family income, this had implications for our sample size which was small. As many of the condition × group interactions did not emerge significantly, and instead we had to rely on post‐hoc simple t tests to explore our hypotheses, it is likely that we were under‐powered to detect these more subtle effects. Related to this, we did not find similar effects for fearful faces even though these are often used to elicit attention bias scores in the anxiety literature (Dudeney et al., 2015). Our data thus require replication in independent samples particularly in relation to the absence of subtype or severity differences.

In summary, this study examined processes of attention control in a sample of maltreated adolescents relative to matched non‐maltreated peers. This study found that maltreatment was associated with general difficulties of attention control, even in the absence of threat‐related or emotional stimuli. These processes may contribute to the emergence of psychiatric disorders and other difficulties for those exposed to maltreatment, and may provide important insights for the development of targeted preventative approaches to improve the life‐long wellbeing for this vulnerable population. Further research of these and other relevant neurocognitive processes that may be affected by maltreatment and contribute to this vulnerability are critical to the development of such interventions.

## NOTE

1 Development of the MacBrain Face Stimulus Set was overseen by Nim Tottenham and supported by the John D. and Catherine T. MacArthur Foundation Research Network on Early Experience and Brain Development. Please contact Nim Tottenham at tott0006@tc.umn.edu for more information concerning the stimulus set
